# Post-transcriptional regulation of DEAD-box RNA helicases in hematopoietic malignancies

**DOI:** 10.1016/j.gendis.2024.101252

**Published:** 2024-02-28

**Authors:** Jiankun Fan, Zhigang Li, Li Pei, Yu Hou

**Affiliations:** a Institute of Life Sciences, Chongqing Medical University, Chongqing 400016, China; b Department of Hematology, Southwest Hospital, Third Military Medical University (Army Medical University), Chongqing 400038, China

**Keywords:** DEAD-box RNA helicase, Hemopoietic system, Post-transcriptional regulation, Ribosomes assembly, RNA alternative splicing

## Abstract

Hematopoiesis represents a meticulously regulated and dynamic biological process. Genetic aberrations affecting blood cells, induced by various factors, frequently give rise to hematological tumors. These instances are often accompanied by a multitude of abnormal post-transcriptional regulatory events, including RNA alternative splicing, RNA localization, RNA degradation, and storage. Notably, post-transcriptional regulation plays a pivotal role in preserving hematopoietic homeostasis. The DEAD-Box RNA helicase genes emerge as crucial post-transcriptional regulatory factors, intricately involved in sustaining normal hematopoiesis through diverse mechanisms such as RNA alternative splicing, RNA modification, and ribosome assembly. This review consolidates the existing knowledge on the role of DEAD-box RNA helicases in regulating normal hematopoiesis and underscores the pathogenicity of mutant DEAD-Box RNA helicases in malignant hematopoiesis. Emphasis is placed on elucidating both the positive and negative contributions of DEAD-box RNA helicases within the hematopoietic system.

## Introduction

Hematopoietic stem cells (HSCs) exhibit the remarkable ability for long-term self-renewal and can differentiate into various lymphocytes and myeloid cells, ensuring a sustained production of mature blood cells.^1^ Typically, the majority of HSCs remain in a quiescent state, while a small subset enters the cell cycle to orchestrate orderly proliferation, differentiation, and self-renewal within the bone marrow. This process ensures the continuous and dynamic production of mature blood cells, which are subsequently released into the circulation.^2^ This intricately regulated and complex process operates at multiple levels and stages, precisely and rapidly controlling protein expression by governing mRNA localization, degradation, and alternative splicing, with RNA serving as the central focus.^3^, ^4^, ^5^

However, the disruption of this delicate regulatory balance leads to leukemic transformation, marked by the emergence of leukemic stem cells. Such a disturbance escalates the likelihood of malignant hematopoiesis, which is distinguished by atypical or hindered differentiation, unregulated self-renewal, and rampant proliferation.^4^ Compelling evidence already exists, suggesting that early hematopoietic stem cells may act as precursors to leukemia stem cells.^6^^,^^7^ Consequently, RNA-centric post-transcriptional regulation is posited to play a crucial role not only in normal hematopoiesis but also in hematologic malignancies.^8^ Importantly, the DEAD-Box RNA helicase family, pivotal in post-transcriptional control, is garnering increasing recognition for its significance within the hematopoietic framework.

The DDX helicase family represents the largest superfamily 2 (SF2) of helicases in eukaryotic cells.^9^^,^^10^ Each member of this family shares a highly conserved amino acid sequence known as Asp-Glu-Ala-ASP (D-E-A-D) and possesses core domains facilitating ATP hydrolysis and RNA unwinding.^11^^,^^12^ Despite variations in ATPase and RNA helicase activities among different DEAD-box RNA helicases arising from complex N-terminal and C-terminal regions, their most fundamental function remains RNA helicase activity.^13^, ^14^, ^15^ This activity is indispensable for their involvement in supporting macromolecular protein complexes, such as the cellular spliceosome and ribosome.^16^

**Figure 1:**
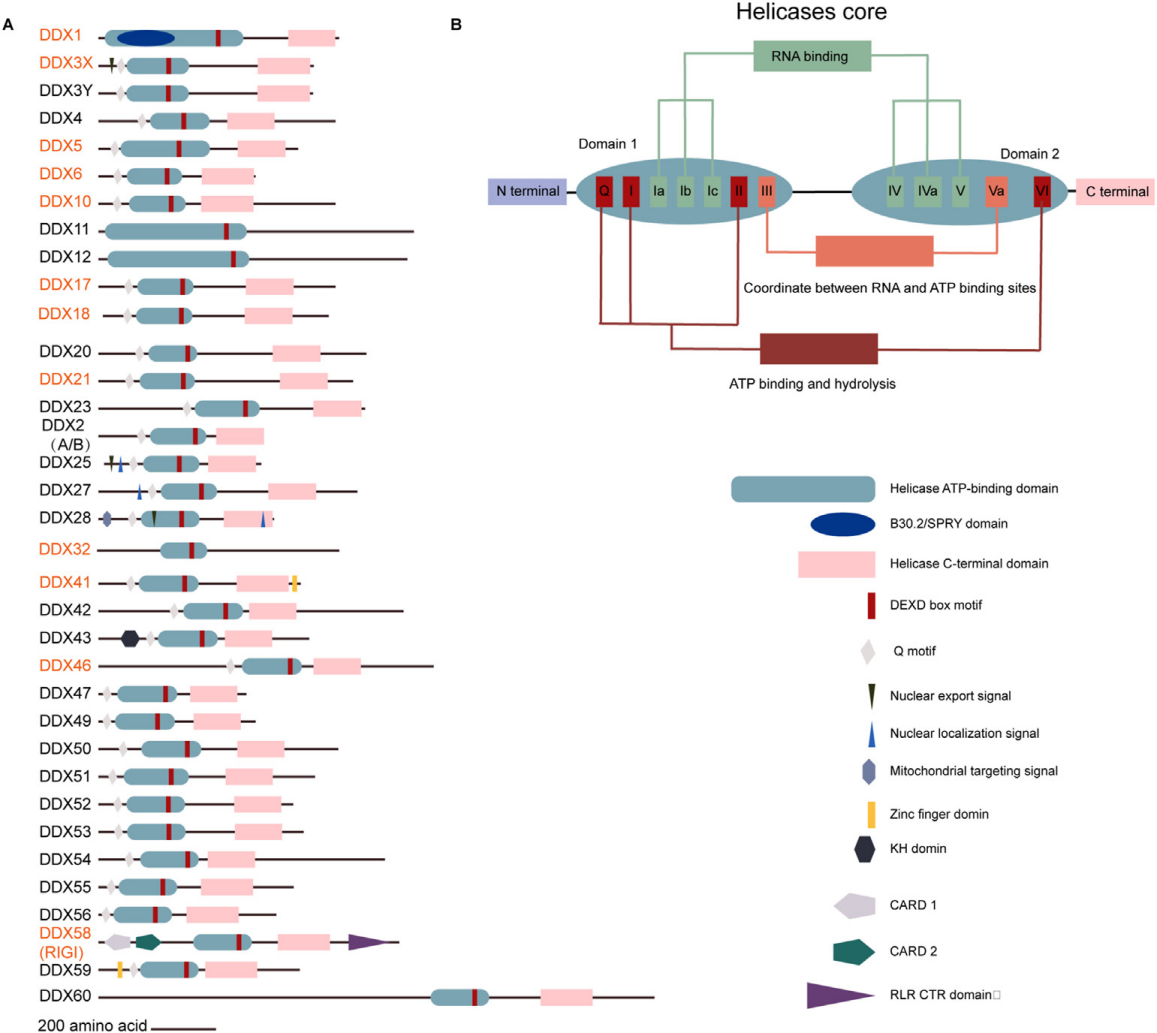
Protein schematics for DEAD-box helicase proteins along with indicated known functional domains and motifs. **(A)** The DEAD-box helicase protein family. Genes previously documented to be associated with the hemopoietic system are highlighted in orange text. **(B)** The helicase core of DEAD-box proteins consists of two spherical domains, namely domains 1 and 2, encompassing a total of 12 motifs with functions related to ATP hydrolysis, RNA binding, and coordination of RNA and ATP binding sites. Consequently, the diverse N-terminal and C-terminal regions contribute complex specific functions through interactions with other regulatory molecules in the capacity of DEAD-box RNA helicases.

The DEAD-box RNA helicase family is defined by 12 highly conserved motifs: Q, I, Ia, Ib, Ic, II, III, IV, IVa, V, Va, and VI, forming two spherical domains (domains 1 and 2) that constitute the helicase cores (Fig. 1B).^17^ Each spherical domain consists of five β chains arranged in an α helix, mimicking the folding pattern observed in the bacterial RecA proteins superfamily.^18^ Motifs Q, I, II, Ia, Ib, Ic, and III constitute spherical domain 1, contributing to three distinct functions: ATP hydrolysis and binding (Q, I, and II), RNA binding (Ia, Ib, and Ic), and coordination of RNA and ATP binding (III).^18^^,^^19^ Notably, motif II includes the conserved amino acid sequence D-E-A-D.^20^

Spherical domain 2, comprising motifs IV, IVa, V, VI, and Va, can be subdivided into three functions: RNA binding (IV, IVa, and V), ATP hydrolysis and binding (VI), and coordination of ATPase and unwinding activity (V). The ATP-binding fissure between these spherical domains facilitates efficient ATP binding and hydrolysis.^21^ These highly conserved core domains govern the binding of DDX helicases to their substrates—proteins, RNA, or DNA.^20^^,^^22^

Within these relatively conserved motifs, the amino acid sequence varies, but a conserved phenylalanine residue upstream of motifs Q and the D-E-A-D amino acid sequence are consistent in all family members, serving as a defining characteristic.^23^^,^^24^ Additionally, the family members possess complex and functional N-terminal and C-terminal regions containing varying lengths, ranging from a few to several hundred amino acids.^22^^,^^25^ These variable regions are believed to confer specificity and complexity to the function of DDX helicases, often associated with activity regulation, modification of proteins and enzymes, protein interactions, and specific functions.^3^^,^^15^ Given their usual RNA helicase activity, members of the DDX helicase family play a central role in diverse cellular RNA metabolism processes—from post-transcriptional regulation to translational initiation (Fig. 1A).^9^^,^^26^

In the hematopoietic system, HSCs undergo regulation by both intrinsic and extrinsic factors to maintain a delicate equilibrium between self-renewal and differentiation. These cells exhibit rapid adaptability to changes in the microenvironment triggered by external signals.^27^ The precise regulation of HSC proliferation and differentiation encompasses multiple levels of control. Extensive research has illuminated the crucial role of post-transcriptional regulation in the hematopoietic process.^28^^,^^29^ This RNA-centric regulation provides a precise and expeditious means for cells to finely adjust protein expression levels. This is achieved by modulating mRNA alternative splicing, localization, storage, and degradation, synergistically interacting with existing transcription networks within cells.^4^^,^^30^ DDX helicases emerge as key contributors to RNA processing, unwinding RNA structures. However, their functional diversity is attributed to the highly variable N-terminal and C-terminal regions.^5^^,^^11^

The hallmark characteristics of hematologic malignancies include chromosomal abnormalities and gene mutations, pivotal in the progression of the disease.^31^ Additionally, with the increasing availability of sequencing data from clinical cases of malignant tumors, it has been noted that mutations in specific genes of the DEAD Box protein family may lead to severe hematological malignancies by disrupting normal post-transcriptional regulatory mechanisms.

The significant role of the DDX helicase family in post-transcriptional regulation has prompted a growing body of research exploring their functions and regulatory mechanisms in the hematopoietic system. These investigations delve into their impact on diverse processes, including protein synthesis, alternative splicing, mRNA decay, and ribosome biogenesis and assembly (Table 1). Despite the wealth of individual studies, a comprehensive review addressing the collective role of the DDX family in the hematopoietic system is notably absent. This review aims to fill this gap by providing a systematic summary of the varied functions of DEAD-box helicases in normal, disordered, and malignant hematopoiesis. Additionally, it delves into an in-depth discussion of the regulatory mechanisms governing the activities of these helicases within the hematopoietic system.

**Table 1:** The DEAD-box RNA helicases with key roles in the hematopoietic system.

Classification	Gene	Mechanism	Hematologic phenotype	Reference
Ribosomes assembly	Ddx3X	Promotes the translation of the target transcript, and increases the formation of 80S complex in DCBCL	Inhibit hematopoietic disease DCBCL	32
	Ddx10	Process 27S ribosomes into mature 25s ribosomes and assembles ribosomes by rRNA processing in eukaryotic cells	Ddx10 forms a fusion gene with NUP98 (NUP98-Ddx10) contributing to the progression of leukemia	33
	Ddx18	Promotes p53-dependent cell cycle arrest in zebrafish	Ddx18-E76del exerts a dominant-negative effect on myelopoiesis	34
	Ddx21	Interacts with amino terminal enhancer of split (AES) to affect snoRNA/RNP production in AML	Ddx21 knockout decreases leukemia self-renewal potential *in vitro* and delays the progression of leukemia *in vivo*	35,36
	Ddx41	Is essential for snoRNA processing, ribosome assembly and protein expression	Ddx41 single-allele mutation induces hematologic phenotype of MDS in elderly mice	37
RNA alternative splicing	Ddx5/17	Makes RNP remodel, disarranges the distribution of splicing factors on a subset of transcripts, indirectly affects pre-mRNA splicing patterns in K562 cell lines	Ddx5 depletion improved AML treatment outcomes	38,39
	Ddx41	Controls to a large number of selective splicing events and affects mRNA metabolism, DNA damage and cell cycle	Ddx41 knockout in HSCs inhibits the normal development of HSCs	40
	Ddx46	Interacts with U2snRNP and catalyzes RNA–RNA rearrangements and RNP remodeling	Ddx46 mutation may affect the maintenance and/or differentiation of zebrafish HSCs	41
RNA decay and storage	Ddx6	Interacts with ATAXIN2, and inhibit mRNA translation in the early stage of megakaryocyte formation	Ddx6 regulate megakaryopoiesis in primary human megakaryoblasts.	42,43
Transcriptional regulation	Ddx1	Interacts with Azin1 to affect the expression of a variety of hematopoietic regulatory factors in the nucleus	Ddx1 knockout reduces bone marrow failure and induces HSC depletion	44
Inflammatory signaling pathway	Ddx58	Inhibits the activation of Akt-mTOR axis promoted by Src in AML	Depletion of Ddx58 in zebrafish inhibits the formation of HSPCs	45,46
Mechanism unclean	Ddx32		Associated with acute lymphocytic leukemia	47

AML, acute myeloid leukemia; DLBCL, diffuse large B cell lymphoma; HSC, hematopoietic stem cells; HSPCs, hematopoietic stem and progenitor cells.

### DDX helicases regulate protein synthesis through ribosomes assembly in normal and malignant hematopoiesis

Ddx3X is situated on the X chromosome, while its homologous gene Ddx3Y, residing on the Y chromosome, shares a 92% conserved protein sequence.^48^ In normal adult tissues, Ddx3Y is primarily expressed in the testis, whereas Ddx3X exhibits transcriptional activity across various tissues.^49^ Generally, Ddx3X facilitates the translation of target transcripts by binding to eIF3. This interaction enhances the association between the 60S subunit and 40S subunits, culminating in the formation of the 80S complex.^50^ Ddx3X demonstrates functional diversity across different tumor types and diseases. It can act both as a tumor inhibitor and a promoter of tumor generation. An examination of an extensive collection of clinical specimens from lymphoma patients indicated that mutations in Ddx3X (R475 and R534), predominantly found in the MHG DLBCL subtype and also observed in medulloblastoma, negate the helicase activity. Cell lines harboring concurrent mutations, R475C and R528C — the most frequently occurring alterations in Ddx3X — or Ddx3X in combination with MYC, demonstrate dominant-negative effects, thereby gaining a proliferative edge. Moreover, the study underscores that the R475C mutation in Ddx3X triggers endoplasmic reticulum (ER) stress. While definitive evidence for the tumor-suppressive role of Ddx3X was not established, the researchers suggested that the mutations in Ddx3X could further reduce global protein synthesis mediated by MYC. This reduction potentially allows cells to withstand the elevated protein synthesis triggered by MYC during the onset of cancer.^32^ Furthermore, it has been reported that ectopic expression of Ddx3Y in fully transformed lymphoma cells increases overall protein translation.^51^ These studies collectively underscore the significant role of Ddx3X/Y in regulating protein synthesis in MYC-driven lymphomas.

Ddx10 is situated on chromosome 11 and was initially identified through cDNA screening in ataxia telangiectasia.^52^ Its association with ribosome assembly in eukaryotic cells has been well-established. Moreover, the functions of Ddx10 closely resemble those of the SPB4 and DRS1 proteins in yeast, both implicated in the processing that converts 27S into mature 25S rRNA.^53^ Subsequent investigations have revealed that Ddx10 forms a fusion gene with NUP98 (NUP98-Ddx10).^54^ This fusion significantly impacts protein synthesis by disrupting normal mRNA transport and selective ribosomal assembly. Consequently, it plays a crucial role in the progression of leukemia.^33^

Ddx18, also known as Has1p in yeast, plays a crucial role in ribosomal RNA processing and has been observed to interact with several ribosomal proteins, including RPS3, RPS3a, RPS6, RPS14, and RPS19.^55^ Ddx18 has been recognized as a crucial gene linked to hematopoiesis due to the insertion of allele hi1727, which disrupts the gene encoding Ddx18. Deletion of Ddx18 results in the loss of myeloid and erythroid cells due to p53-dependent G1 cell-cycle arrest and increased apoptosis. Concurrently, the authors identified four non-synonymous sequence mutations of Ddx18 in human leukemia samples by using next-generation sequencing technology. Among these mutations, Ddx18-E76del failed to rescue hematopoiesis in Ddx18-deleted embryos, classifying this non-synonymous sequence mutation as a dominant-negative allele.^34^Although the presence of the mutant allele (Ddx18-E76del) has been confirmed in human leukemia cases, a direct causal link between Ddx18 deletion or mutation and hematopoietic system regulation through ribosome assembly remains elusive. However, based on research conducted on Ddx18 in the hematopoietic system and ribosome assembly, the authors propose that the Ddx18 mutant may induce a relative ribosomal protein-deficient state. This could result in the upregulation of RPL11, inducing ribosomal stress and subsequently stabilizing p53.^55^^,^^56^ Further investigations are required to ascertain the extent to which the relative ribosomal protein deficiency induced by the Ddx18 mutant contributes to the observed phenotype.

Ddx21, a nucleolar helicase situated in the nucleus of eukaryotic cells, plays a pivotal role in cellular processes.^57^ Knocking out Ddx21 in tumor cells leads to a reduction in ribosomal content, including rRNA and ribosomal proteins. The rate of mRNA-to-protein translation is lowered as a result, thereby impeding the growth of tumor cells.^36^^,^^58^ In individuals with acute myeloid leukemia (AML), Ddx21 interacts with the amino-terminal enhancer of split (AES), influencing the production of small nucleolar RNAs (snoRNAs) and ribonucleoprotein (RNP).^35^ The deficiency of snoRNA results in diminished protein synthesis and reduced cell volume in leukemia cells, which diminishes their self-renewal potential *in vitro* and retards the progression of leukemia *in vivo*.^35^ Furthermore, Ddx21 has been identified as a prognostic indicator in breast cancer through gene expression profiling. In BRCA1/2-wild-type breast cancer cells, snoRNA activates PARP-1, leading to ADP-ribosylation of Ddx21, thereby enhancing ribosome biogenesis and cell proliferation. Mutations in the ADP-ribosylation sites on Ddx21 result in PARP-1 depletion and decreased Ddx21 at the rDNA locus, leading to a reduction in cellular ribosomal content and ultimately inhibiting protein translation.^35^^,^^36^

Ddx41 is associated with innate immunity, inflammation, R-loop metabolism, and RNA alternative splicing.^59^^,^^60^ In myelodysplastic syndrome (MDS) with a Ddx41 mutation, the most frequent germline mutation is a frameshift mutation with aspartic acid (D) 52 or D140, resulting in an inactive Ddx41 protein and dysfunction. Approximately 50% of these patients also acquire a somatic mutation at arginine 525 to histidine (R525H).^37^ Many affected individuals have a germline single-allele frameshift mutation, and the remaining cells acquire somatic mutations of the Ddx41 allele, with the most common being the mismatch R525H.^37^ Research utilizing a conditional knockout mouse model has established that Ddx41 mutations (R525H) contribute to the progression of MDS. This process is mediated not only by defects in ribosome translation but also by irregular snoRNA processing.^40^^,^^61^ Nevertheless, hematopoietic progenitor cells (HPCs) carrying biallelic frameshift mutations undergo cell cycle arrest and apoptosis, ultimately leading to bone marrow failure.^62^ Further investigations have elucidated that Ddx41 plays a pivotal role in the snoRNA processing within hematopoietic stem and progenitor cells (HSPCs). Disruption of Ddx41-dependent snoRNA processing leads to ribosomal anomalies and diminished protein synthesis, subsequently triggering cell cycle arrest and apoptosis in the proliferating HSPCs.^40^^,^^62^ Despite HSCs predominantly being in a quiescent state, which provides protection against the injury induced by reduced protein synthesis associated with ribosome defects caused by Ddx41 mutations, proliferative precursor cells such as HPCs and erythroid progenitor cells are particularly sensitive to translation defects, This observation aligning with previous reports in zebrafish.^63^

Indeed, DDX helicases play significant roles in maintaining balance of protein synthesis, a critical aspect of proper cellular function. Dysregulation in protein synthesis can result in various diseases, including hematopoietic malignancies. Within the hematopoietic framework, DDX helicases are indispensable for modulating protein synthesis via diverse pathways, including ribosome assembly, mRNA splicing, and snoRNA processing. Deletion or mutation of these proteins can disrupt the balance of protein synthesis, leading to various malignant blood diseases, such as MDS and leukemia. Consequently, further exploration of the intricate regulatory mechanisms of protein synthesis by DDX helicases and their pivotal role in sustaining hematopoietic equilibrium is imperative.

### DDX helicases regulate RNA alternative splicing in normal and malignant hematopoiesis

RNA alternative splicing is a crucial regulatory mechanism during gene expression, removing non-coding sequences from immature RNA to produce mature mRNAs.^64^^,^^65^ The splicing machinery comprises large nucleoprotein complexes known as spliceosomes, which include small nuclear RNAs (snRNAs) and splicing factor proteins.^66^ Mutations in splicing factors are acknowledged as significant contributors to myeloid malignancy.^67^^,^^68^ Notably, spliceosome mutations affecting RNA splicing factors are present in over 50% of patients with MDS.^69^ Additionally, certain DDX helicases homologous to yeast splicing factors have been confirmed to play an important role in regulating RNA alternative splicing in hematopoietic malignancies.^30^^,^^70^

Ddx5 and Ddx17 exhibit the highest homology among members of the DDX family. Their helicase core regions display up to 90% sequence homology, encompassing functions related to RNA, ATP binding, and hydrolysis. Consequently, Ddx5 and Ddx17 share similar functions to some extent. For instance, both helicases regulate the nuclear transportation of β-catenin, thereby contributing to tumorigenesis and tumor progression.^71^ Additionally, they both demonstrate RNA annealing activity and catalyze the rearrangement of RNA secondary structure.^39^ However, Ddx5 and Ddx17 possess unique functions attributed to distinct N-terminal and C-terminal sequences in the cell.

Ddx5 was initially identified as part of a large neuronal RNA-protein complex that regulates pre-mRNA splicing in neuronal cells.^72^^,^^73^ In the human AML cell line K562, two coordinated splicing factors, hnRNPA1 and Ddx5, were identified by analyzing various pre-mRNA alternative splicing patterns, RNA binding sites, and nearby RNA structures.^71^ Ddx5 is involved in the regulation of variable alternative splicing, showing significant overlaps in splicing targets with hnRNPA1.^39^ Knockdown of Ddx5 results in RNP remodeling, redistribution of splicing factors on a subset of transcripts, and indirect effects on pre-mRNA splicing patterns. Furthermore, Ddx5 depletion has enhanced AML treatment outcomes by inducing cell cycle arrest, apoptosis, downregulation of glucose metabolism-related genes, and increasing ROS levels.^39^

Additionally, Ddx5 and Ddx17 play a role in regulating male and female sex hormone signaling pathways through RNA alternative splicing.^39^ Indeed, depletion of Ddx5/Ddx17 induces the production of a SMRT alternative splicing variant that is decreased through the NMD pathway, resulting in decreased SMRT protein. Moreover, Ddx5 and Ddx17 modulate AR and ERa proteins by regulating GSK3b alternative splicing. These findings suggest that Ddx5 and Ddx17 play a broad role in controlling steroid hormone signal pathways, influencing both upstream and downstream of hormone receptors. However, research on how Ddx5 and Ddx17 regulate the hematopoietic system through alternative splicing is currently limited.^74^ More papers are needed to explore the detailed mechanism of Ddx5/Ddx17 in regulating alternative splicing in hematopoietic system.

Ddx41 serves as a critical regulator of the hematopoietic system through its involvement in RNA alternative splicing. By interacting with spliceosome components, Ddx41 plays a role in the intricate regulation of pre-mRNA alternative splicing.^63^ Mutations in Ddx41 (sa14887) are implicated in the onset of hematological malignancies, primarily due to abnormal alternative splicing provoked by the mutant Ddx41 (sa14887).^59^ The deletion of Ddx41 results in the misexpression and aberrant alternative splicing of cell cycle genes within erythroid progenitor cells harboring the Ddx41 mutation (sa14887).^63^ Moreover, cells with the Ddx41 mutation manifest extensive DNA damage responses and cell cycle arrest, a situation that can be partially remedied by inhibiting these factors, thereby alleviating the anemia associated with the Ddx41 mutation.^75^^,^^76^ In addition, the absence of Ddx41 triggers a plethora of selective splicing events, such as exon skipping, intron retention, and alternative 5′ or 3′ splicing.^62^^,^^77^ Pathway analysis reveals the suppression of various cytokine pathways, such as SMC5 and STAT1a, which are affected by these abnormal alternative splicing events, These events are primarily enriched in mRNA metabolism, DNA damage, and cell cycle pathways.^77^ Subsequently, Ddx41 knockout results in reduced proliferation, myeloid loss, and differentiation defects *in vivo*.^78^ Significant variations in the expression of genes associated with differentiation and proliferation are evident when comparing Ddx41 knockout HSCs with their wild-type counterparts. This observation suggests that the absence of Ddx41 in HSCs hampers their typical development, diverging from the precancerous phenotype commonly observed in hematopoietic malignancies.^61^^,^^63^ Ddx41 knockout in HSCs results in a multitude of aberrant splicing events, including exon skipping and intron retention. Specific instances, such as intron retention in Abcf1, Imp 4, and Emg1, exemplifying these abnormalities.^77^^,^^79^ The widespread occurrence of abnormal splicing events in Ddx41 knockout HSCs has repercussions on proteins involved in diverse biological pathways. Serving as a pivotal regulator of alternative splicing, Ddx41 influences the nonsense-mediated decay induced by alternative splicing, thereby modifying the proportion of protein isomers. Ultimately, Ddx41 assumes a critical role in essential biological processes, including ribosome biogenesis, translation initiation, and mRNA processing.^61^ These functions contribute to the abnormal translated products that promote the Ddx41 knockout phenotype, causing inhibition of proliferation and differentiation of HSCs.

Yeast DEAD-box protein 46, also known as Prp5, plays a pivotal role in distinct stages of alternative splicing by facilitating RNA–RNA rearrangements and RNP remodeling.^80^ In zebrafish with a Ddx46 mutation, characterized by the alteration of Prp5, the accumulation of un-spliced Gata1a mRNAs is observed. This aberrant pre-mRNA alternative splicing leads to a reduction in Gata1a expression and the inhibition of erythropoiesis. These findings suggest that the Ddx46 mutation-induced irregularities in pre-mRNA alternative splicing may impact the homeostasis, differentiation, and proliferation of hematopoietic stem cells in zebrafish.^41^ However, there is currently no documented association between Ddx46 mutations and hematopoietic malignancies such as CLL or MDS in patients who have undergone whole-exome sequencing. Nevertheless, it remains plausible that Ddx46 mutations could elevate the susceptibility to hematopoietic malignancies.

### DDX helicases regulate RNA decay and storage through P-bodies in normal and malignant hematopoiesis

Ddx6, endowed with intrinsic helicase activity, plays a pivotal role in the formation of processing bodies (P-bodies) in pluripotent cells. P-bodies, membrane-less organelles, are created through the assembly of RNA and nearby RNA-binding proteins (RBPs) into RNP granules, facilitating phase separation.^42^ They function as repositories for untranslated mRNAs, segregating them from the translational program. Ddx6 actively maintains P-body numbers and represses the translation of Ddx6-bound mRNAs within P-bodies. Notably, Ddx6 selectively represses key transcription factors and chromatin regulators associated with pluripotency mRNAs, such as ZIC3 and MSL2, without acting as a global repressor of translation.^81^ Moreover, Ddx6 facilitates the degradation of differentiation-inducing factors, including KLF4, by binding to their respective mRNAs. Within the hematopoietic system, Ddx6 influences the homeostasis of megakaryocytes by directly interacting with ATAXIN2, a regulatory factor highly expressed in CD34+/CD41+ megakaryocytes. This interaction inhibits mRNA translation during the early stages of megakaryocyte formation.^43^ The interaction between Ddx6 and ATAXIN2 regulates megakaryopoiesis in primary human megakaryoblasts. The Ddx6-PABP complex induces miRNA-mediated mRNA decay, whereas ATAXIN2-associated Ddx6/PABP-bound mRNA is only silenced and not degraded within P-bodies.^42^^,^^43^ These findings suggest that Ddx6 may control relative mRNA decay or storage to regulate the hemopoietic system.

### Other regulatory mechanisms

The DDX protein family comprises hypothetical RNA helicases.^82^ As previously noted, proteins within the DDX family play a regulatory role in the alternative splicing of pre-mRNA within HSPCs, thereby influencing both normal hematopoiesis and certain malignant diseases.^83^^,^^84^ Moreover, DDX helicases exert their influence on the development of the hematopoietic system by directly binding to and unwinding corresponding DNA, thereby modulating transcriptional regulation.^11^^,^^85^ For example, in normal hematopoiesis, the RNA-edited Azin1 protein interacts with Ddx1 and directly governs the expression of various hematopoietic regulators in the nucleus, promoting the differentiation of HSPCs.^44^^,^^86^ When the aberrant RNA-edited Azin1 protein binds to Ddx1, it alters the chromatin distribution of Ddx1, impacting the expression of a variety of hematopoietic regulators in the nucleus and ultimately impairing the differentiation of HSPCs.^44^ However, the detailed mechanisms through which Ddx1 regulates the expression of various hematopoietic regulators in the nucleus remain obscure.^87^^,^^88^ Given the RNA unwinding functional domains possessed by most DDX helicases, enabling them to unravel complex RNA structures, ongoing studies are shedding light on this unwinding process. Consequently, it is plausible that additional members of the DDX family will be investigated for similar functions within the normal hematopoietic system.

Ddx58 plays a pivotal role in the regulation of the hematopoietic system through the inflammatory signaling pathway. Also known as retinoic acid-induced gene protein I (RIG-I), Ddx58 is indispensable for hematopoiesis. In zebrafish, the depletion of Ddx58 impedesHSPC formation, resulting in a diminished production of inflammatory signals.^45^ Furthermore, Ddx58 has been shown to be crucial for myeloid development and the maintenance of leukemic stem cell stemness.^45^ In AML cells, Ddx58 inhibits the activation of the Akt-mTOR axis promoted by Src, exerting anti-leukemic activity through competitive inhibition of the Src/Akt association.^89^ However, the functions of the DDX helicase family are intricate, and several members, such as Ddx32, exhibit unclear roles in the hematopoietic system. Ddx32 is downregulated in acute lymphocytic leukemia, though the underlying mechanisms remain to be elucidated.^47^ Comprehensive research is warranted to fully comprehend the regulatory role of DDX helicases in the hematopoietic system.

## Conclusion

In summary, the DDX helicase family plays important roles in regulating both normal and malignant hematopoiesis. Several DDX protein mutations, such as those in Ddx10, Ddx18, and Ddx41, have been associated with malignant hematopoiesis (Table 2). Conversely, a subset of studies has highlighted the critical importance of DDX protein deletions in both normal and malignant hematopoiesis, exemplified by Ddx21, Ddx41, Ddx1, and others. The functions of these helicases encompass diverse mechanisms, including RNA alternative splicing, ribosome assembly, and transcriptional regulation. Despite significant progress, the underlying mechanisms remain incompletely understood, necessitating further studies to unravel the molecular intricacies and regulatory networks. In-depth analysis of the interaction modes between proteins and RNA or DNA is imperative for a profound understanding of the DDX family's role in hematopoiesis. Additionally, DDX helicase family proteins present potential targets for artificially modulating the proliferation and differentiation of HSCs, offering promising avenues for the development of targeted treatments in hematological malignancies.

**Table 2:** The mutation hotspots of DEAD-box RNA helicases within the hematopoietic system.

Gene	Mutation	Function region	Dysfunction	Hematopoietic malignancy	Reference
Ddx3X	R475 mutant	C-terminal helicase domain	MYC translocation	DCBCL	32
Ddx10	NUP98-Ddx10	Fusion with NUP98 in helicase ATP binding domain	mRNA transport and ribosomal assembly	Leukemia	33
Ddx18	Ddx18-E76del	Polar residues	Up-regulation of RPL11 and ribosomal stress	MDS/AML	34
Ddx41	R525H	C-terminal helicase domain	snoRNA and protein translation	MDS	40
	sa14887		DNA damage	Anemia	63

AML, acute myeloid leukemia; DLBCL, diffuse large B cell lymphoma; MDS, myelodysplastic syndrome.

## Declaration of generative AI and AI-assisted technologies in the writing process

During the preparation of this work, the authors used “ChatGPT” to enhance language quality and readability. After using this tool/service, the authors have reviewed and edited the content as needed and take full responsibility for the content of the publication.

## Author contributions

Fan and Li contributed equally to the writing of this manuscript. Hou and Pei supervises and edits this review. All authors have read and agreed to the published version of the manuscript.

## Conflict of interests

The authors declare no competing interests.

## Funding

This work was supported by Chongqing Science Fund for Distinguished Young Scholars (No. CSTB2022NSCQ-JQX0032), Chongqing University Innovation Research Group Project (No. CXQT21011), Chongqing Medical University Youth Innovation in Future Medicine (No. W0156), the National Natural Science Foundation of China (No. 82200123), and Natural Science Foundation of Chongqing, China, (No. CSTB2023NSCQ-MSX0280).
